# Hand hygiene practices during meal preparation—a ranking among ten European countries

**DOI:** 10.1186/s12889-023-16222-5

**Published:** 2023-07-10

**Authors:** Octavian Augustin Mihalache, Paula Teixeira, Solveig Langsrud, Anca Ioana Nicolau

**Affiliations:** 1 Dunarea de Jos University of Galati, Faculty of Food Science and Engineering, Domnească Street 111, 800201 Galati, Romania; 2 Universidade Católica Portuguesa, CBQF - Centro de Biotecnologia e Química Fina – Laboratório Associado, Escola Superior de Biotecnologia, Rua Diogo Botelho 1327, 4169-005 Porto, Portugal; 3 Nofima, Norwegian Institute of Food, Fisheries and Aquaculture Research, Osloveien 1, N-1430 Ås, Norway

**Keywords:** Hand cleaning, Hygiene practice, Hand wash, Soap, Food preparation, Raw chicken

## Abstract

**Background:**

The aim of this paper was to map consumers’ food hygiene practices from 10 European countries and evaluate which demographic groups are more likely to be exposed to foodborne pathogens and establish a ranking of adherence to food hygiene practices in 10 European countries.

**Methods:**

The research design consisted of a cross-national quantitative consumer survey regarding food safety and hygiene practices during meal preparation (SafeConsume project) and was conducted in ten European countries (France, Denmark, Germany, Greece, Hungary, Norway, Portugal, Romania, Spain and UK). The survey questions were based on recommended hand hygiene practices and on observed practices from a field study performed in 90 European households from six of the countries covered by the survey (France, Hungary, Norway, Portugal, Romania, and UK).

SPSS Statistics 26 (IBM Software Group, Chicago, IL) was used for the descriptive and regression analyses of the data. Regression analyses were used to check the relation between demographic characteristics, country of origin and self-reported hand hygiene practices.

**Results:**

According to the regression models, families with elderly members aged over 65 showed a higher tendency to follow proper hand washing practices compared to families without elderly members. Meanwhile, families with children under the age of 6 reported being up to twice as likely to wash their hands at critical moments compared to families without children.

Overall, taking into consideration the likelihood of washing hands after touching raw chicken and the percentages scores for proper hand cleaning methods and key moments for hand washing, the rank of the countries regarding proper hand hygiene practices was the following: Denmark, Greece, Norway, Romania, Hungary, Germany, UK, Portugal, France, and Spain.

**Conclusions:**

Information and education should point both at the key moments as suggested by the Royal Society for Public Health (RSPH) and the International Scientific Forum on Home Hygiene (IFH) and safe practices.

Public health burden generated by improper hand washing may be significantly reduced if education is targeted on consumers’ behaviour and practices.

**Supplementary Information:**

The online version contains supplementary material available at 10.1186/s12889-023-16222-5.

## Introduction

In 2021, the European Food Safety Authority (EFSA) and the European Centers for Disease Control and Prevention (ECDC) reported 4,005 foodborne outbreaks, and 32,543 foodborne illnesses. In the same year, a total number of 2,495 hospitalisations and 31 outbreak-related deaths were reported. Inadequate consumer food preparation practices (e.g., cross-contamination, insufficient heat treatment) in the household environment were the most commonly reported cause of outbreaks [1]. The number of foodborne diseases at the domestic level is underreported and lacks consistency for a number of reasons. Many countries do not report household outbreaks [1] and most probably illness acquired in households often appears sporadically and people do not always seek medical help.

Hand washing with water and soap has proven to be an effective method in preventing/reducing the risk of infectious diseases. Several studies revealed that hand washing with soap reduces the risk of diarrhoeal disease by 23 – 48% [2, 3], and the risk of respiratory infections by 21 – 23% [4, 5]. Research revealed that consumers who wash hands before preparing food reported less foodborne illnesses than those who did not [6]. Hand hygiene is even of greater importance for vulnerable consumers such as the elderly, children, and pregnant women [7].

To prevent the risk of foodborne illness, official bodies such as the Centers for Disease Control and Prevention (CDC) and the World Health Organisation (WHO) recommend washing hands with warm water and soap for 20 s prior to beginning food preparation and at key moments (e.g., after wiping the counter or other surfaces with chemicals, touching the garbage, using the toilet, etc.) [8, 9]. Kendall et al., [10] found a good correlation between observed practices and answers recived when questioning people about washing hands, but only if rinsing hands is also considered as being a washing hands procedure. Observational studies indicate that during cooking, many consumers do not follow the recommended hand washing procedure but just rinse their hands [11–14] and state reasons like: “*My schedule is very tight in the evening. You know, during that time, washing hands for 20 s or checking the cooking temperature is just difficult*.” [15]. These data indicate the need for further investigations regarding consumers’ hand hygiene practices since it is a key element for the prevention of foodborne diseases.

There are reports about consumer hygiene in the kitchen from many countries, but they are scattered in time and place [16–18]. For the current observational and self-reporting studies regarding hand washing during food preparation besides country differences and demographic differences, there is a lack of studies with more than one country. Most of the time observational studies have a lower number of participants than self-reported studies and this is why surveys prevail since risk assessments need quantitative data in order to develop advice that is targeted to different cultures and consumer groups [11, 19]. Self-reported hand hygiene studies are important to draw attention to consumers’ behaviour. If consumers think they comply with proper hygiene practices they are inclined to not take into consideration educational campaigns regarding hand hygiene [20].

Thus, by mapping consumers’ self-reported common hand hygiene practices, we will be able to make recommendations accordingly to their practices. Hence, the objectives of this paper were to evaluate which demographic groups are more likely to be exposed to foodborne pathogens and assess consumers’ self-reported hand hygiene practices from 10 European countries. This study is part of the SafeConsume, where previous parts of the material from the project were published, but only including three different consumer groups [11, 14, 21]. The present study is based on a representative collection of households from ten European countries.

## Materials and methods

The research design consisted of a cross-national quantitative consumer survey regarding food safety and hygiene practices during meal preparation (SafeConsume project) and was conducted in ten European countries (France, Denmark, Germany, Greece, Hungary, Norway, Portugal, Romania, Spain and UK). The survey questions were based on recommended hand hygiene practices and on observed practices from a field study performed in 90 European households from six of the countries covered by the survey (France, Hungary, Norway, Portugal, Romania, and UK) [14].

### Survey design and data collection

The questions used in this study are part of a larger survey, which had 165 questions and was conducted through a professional survey provider (Dynata, https://www.dynata.com/) between December 2018 and April 2019. Population sampling was based on the Nomenclature of Territorial Units for statistics level 2 (NUTS2) and the education level. The total number of respondents was 9966. The total number of respondents was 9966, with the number of respondents per 100,000 inhabitants as follows: 17.6 in Denmark, 1.5 in France, 1.2 in Germany, 8.3 in Greece, 10.4 in Hungary, 18.6 in Norway, 9.1 in Portugal, 5.2 in Romania, 2.1 in Spain, and 1.6 in the UK.

All the details referring to the recruitment, methodology applied and anonymization are described by Mihalache et al., [21]. Apart from demographic details, two questions emphasized on: self-reported likeliness of washing hands (the WHO recommended procedure with water and soap) after handling raw chicken (ordinal scale, 1 – no chance/almost no chance, 11 – certain/practically certain) and applying hand hygiene procedures after handling raw chicken (10 hand cleaning procedures coded HCP; nominal scale, yes/no) (answers received from 79% of respondents). The other two questions highlighted self-reported hand hygiene at key moments (six key moments coded KM; nominal scale, yes/no) and general hand hygiene practices (10 hand cleaning procedures coded HCP; nominal scale, yes/no) applied by consumers at home (answers received from all 9966 respondents). We included so many alternatives because usually surveys do not ask details about the washing process and Didier et al., [11] highlighted consumers’ ambiguity regarding hand washing (they mention washing hands, while they are just rinsing hands).

### Statistical analysis and survey reliability

SPSS Statistics 26 (IBM Software Group, Chicago, IL) was used for the descriptive and regression analyses of the data. The normality of the data was evaluated with the Shapiro–Wilk test. The test showed that the data is not normally distributed (*p* < 0.05), hence the Kruskal–Wallis and Chi-squared tests were used for the comparison of data between countries.

The reliability of the survey was acceptable as indicated by Cronbach’s alpha coefficient (α = 0.72). Regression analyses were used to check the relation between demographic characteristics, country of origin and self-reported hand hygiene practices. Ordinal regression was used for the likeliness of washing hands after touching raw chicken, while for the other three questions we computed new variables which included only the correct answers for proper hand cleaning methods and key moments when hand washing should occur. Hence, the questions “*How would you clean your hands after touching raw chicken?”* and “*In general, how do you normally wash your hands when you are at home?”* were computed into “Proper hand cleaning methods after handling raw chicken” and “Proper hand cleaning methods” which included only the following answers: “Wash hands with regular soap (bar or liquid)”, “Wash hands with antibacterial soap” and “Make sure I wash my hands for at least 20 s” [9]. For the question “*In general, when would you normally wash your hands at home?”* we created a variable named “Washing hands after touching a high-risk item” (the correct response for this question was ticking all the answer variants).

The Omnibus Test was used to assess if the regression models significantly improve when compared to the null model (*p* < 0.05). The goodness of fit for the binary models was assessed with the Pearson and Deviance tests, which indicate good model fit when they are not significant at *p* > 0.05 [22], while the assumption of proportional odds or the parallel lines test was used for the ordinal regression models (significant at *p* > 0.05) [23].

## Results

### The socio-demographic profile of respondents

The socio-demographic profile of the respondents from this study was previously provided in Mihalache et al., [24].

### Self-reported hand hygiene practices

**Fig. 1:**
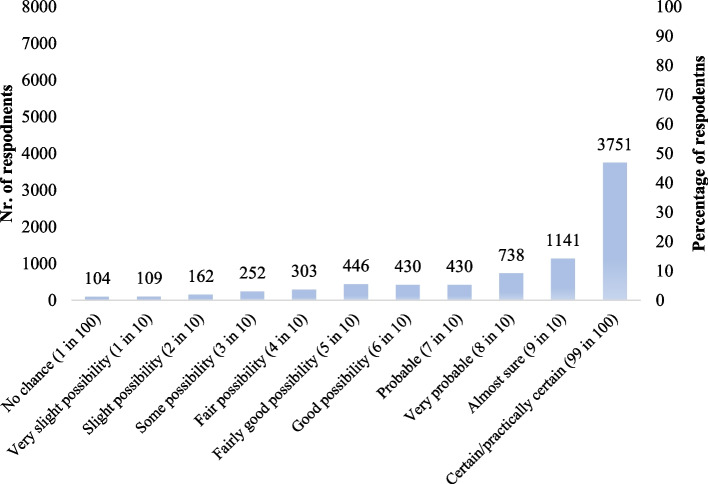
Self-reported likeliness to wash hands after touching raw chicken based on SafeConsume survey

**Fig. 2:**
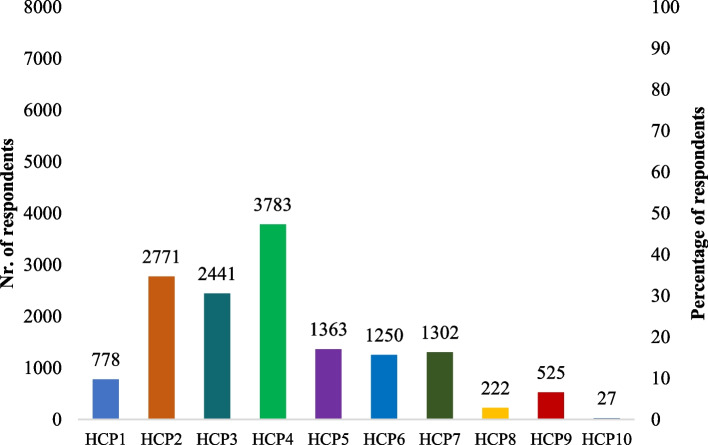
Self-reported hand cleaning methods after touching raw chicken based on the SafeConsume survey; only Yes answers are displayed;; HCP1 = Wash hands with cold water; HCP2: Wash hands with warm water; HCP3: Wash hands under running water; HCP4: Wash hands with regular soap (bar or liquid); HCP5: Wash hands with antibacterial soap; HCP6: Make sure I wash my hands for at least 20 s; HCP7: Dry hands using a paper towel/cloth/kitchen roll; HCP8: Let my hands dry in the air; HCP9: Disinfect my hands with a hand disinfectant (both alcohol-containing hand sanitizers and sanitizers without alcohol); HCP10: I do not wash my hands

**Fig. 3:**
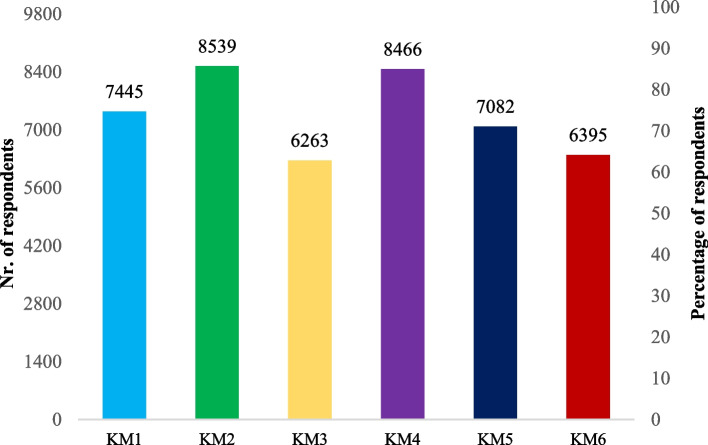
Self-reported hand cleaning methods practiced by respondents after touching a high-risk item based on the SafeConsume survey; only Yes answers are displayed; KM1 = After touching raw meat or eggs; KM2: After going to the toilet; KM3 = After touching or feeding animals; KM4 = After touching something dirty (physical contact with a soiled/contaminated product/surface); KM5 = After mopping up spillages from poultry or eggs; KM6 = After touching a dirty cloth or sponge

**Fig. 4:**
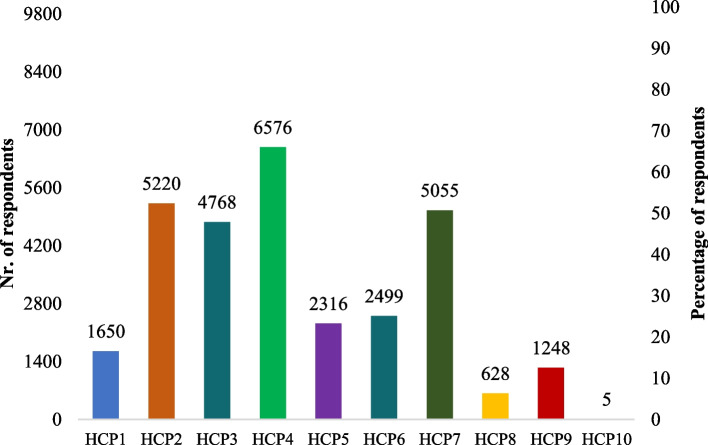
Self-reported general hand cleaning methods practices by respondents based on the SafeConsume survey; only Yes answers are displayed;; HCP1 = Wash hands with cold water; HCP2: Wash hands with warm water; HCP3: Wash hands under running water; HCP4: Wash hands with regular soap (bar or liquid); HCP5: Wash hands with antibacterial soap; HCP6: Make sure I wash my hands for at least 20 s; HCP7: Dry hands using a paper towel/cloth/kitchen roll; HCP8: Let my hands dry in the air; HCP9: Disinfect my hands with a hand disinfectant (both alcohol-containing hand sanitizers and sanitizers without alcohol); HCP10: I do not wash my hands

Figures. 1, 2, 3 and 4 display respondents’ self-reported hand hygiene practices. Almost half of the respondents (47.7%; 3,771/7866) self-reported that they wash their hands after touching raw chicken.

Half of the respondents (48.1%; 3,783/7,866) self-reported to wash hands with soap, however, only 15.1% (1,187/7,866) reported to respect the recommended washing duration of 20 s.

Regarding hand washing after touching a high-risk item, high percentages of self-reported hand washing were noticed after going to the toilet (85.7% 8,539/9966) and after touching something that may harbour pathogens (84.9%; 8,466/9966). Only three quarters (75%; 7,445/9966) wash hands after touching raw meat or eggs.

Overall, more than half of the respondents (6,576/9966; 66%) self-reported to wash hands with water and soap as it is recommended by organizational bodies such as the European Centers for Disease Control and Prevention [25]. Similar to our results, other self-reported studies indicate that 85% of Irish consumers and 66.4% of Canadian consumers know the recommended hand hygiene practices [26, 27].

Based on our survey, a low percentage of respondents wash their hands for at least 20 s (25.1%; 2,499/9966), while half of them shorten the duration of the hand cleaning procedure, either by washing hands for less than 20 s or just by rinsing their hands in running water (47.8%; 4,768/9966). Although this procedure is not among the recommended hand washing practices, it was proven to have an efficacy close to hand washing with warm water and soap (6 – 8% less effective) [28].

### Demographic factors associated with hand hygiene practices

To assess the relation between respondents’ demographic characteristics and hand hygiene practices we used regression analyses regarding hand washing after handling raw chicken, washing hands at key moments, and general hand cleaning methods. Odds ratios (OR) > 1 indicated that respondents are more likely to perform hand hygiene practices, while OR < 1 implied a lower level of compliance to the mentioned practices.

Age was a significant predictor for self-reported hand hygiene practices revealing that older respondents (> 35 years old) were more likely to report proper hand hygiene practices than younger ones (aged < 35 years) (*p* < 0.05; OR = 1.15 – 2.4).

The level of education was also a significant predictor of hand hygiene practices as respondents with middle/high level of education are almost three times more inclined to report adequate hygiene practices at key moments, including raw chicken preparation (*p* < 0.05; OR = 2.82).

Related to inhabitancy, respondents from the urban area were more likely to report key moments when hand hygiene should be applied than respondents from the rural area (*p* < 0.05; OR = 1.3).

Regarding vulnerable groups, pregnant women were less inclined to report proper hand hygiene practices at key moments when hand washing should occur (*p* < 0.05; OR = 0.47 – 0.54).

Members of families with young children (< 6 years old) were two times more likely to wash hands after touching a high-risk item than the members of families without young children (*p* < 0.05; OR = 1.97).

Members of families with elderly members (> 65 years old) were less likely to report proper hand hygiene practices at key moments than the members of families with no elderly (*p* < 0.05; OR = 0.37).

### Hand hygiene practices across 10 European countries

**Fig. 5:**
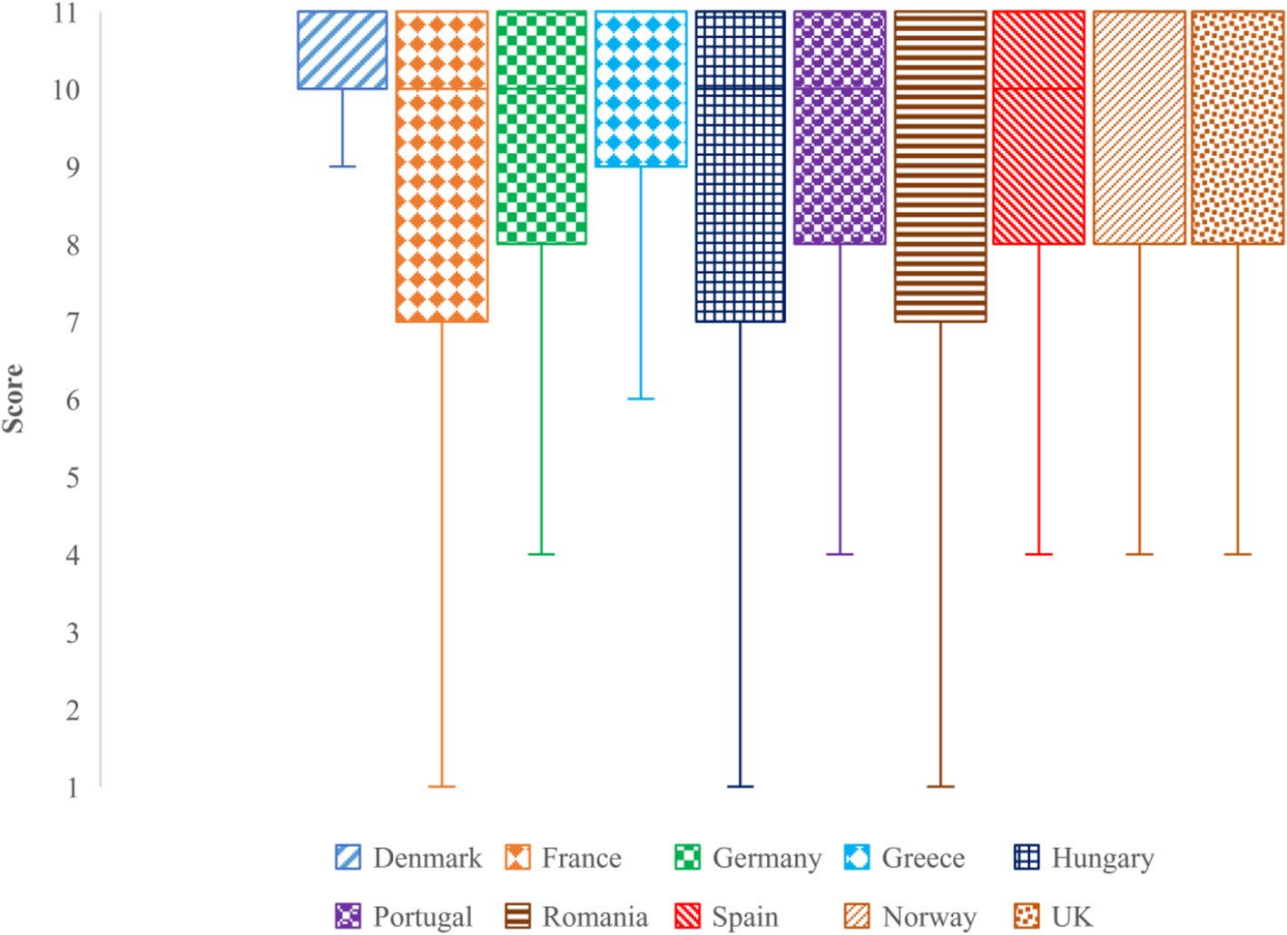
Self-reported likeliness to wash hands after touching raw chicken per country based on the SafeConsume survey

Figure 5 indicates the likelihood of respondents to wash hands after handling raw chicken per country.

The figure shows that all of the boxplots are right-skewed and have the main body of data condensed around high scores (between 7 (probable) – 11 (certain)). Based on self-reported answers, seven countries, France, Germany, Hungary, Portugal, Romania, and Spain, shared the same median (10), while Denmark, Greece, Norway, and UK had a median of 11.

Reported in percentages, the likelihood of respondents to wash their hands after handling raw chicken, more than 60% for the Danish respondents (66.3%; 541/816). Similar results were found for British (57.3%; 525/916), Greek (57.2%; 452/790), and Norwegian respondents (52.3%; 441/844). Low percentages of respondents (< 2%) declaring they would not wash their hands were observed for all the countries. No significant differences for the likeliness of washing hands after touching raw chicken were found between: France – Hungary – Spain, Germany – Portugal – Norway, Germany – Hungary – Romania, Romania – Portugal, and Spain – Romania, indicating similar hand hygiene practices for these countries (*p* > 0.05; Table S1).

Overall, the likelihood to wash hands after manipulating raw chicken from the highest probability to the lowest was in this order: Denmark, UK, Greece, Norway, Portugal, Germany, Romania, France, Hungary, and Spain.

**Fig. 6:**
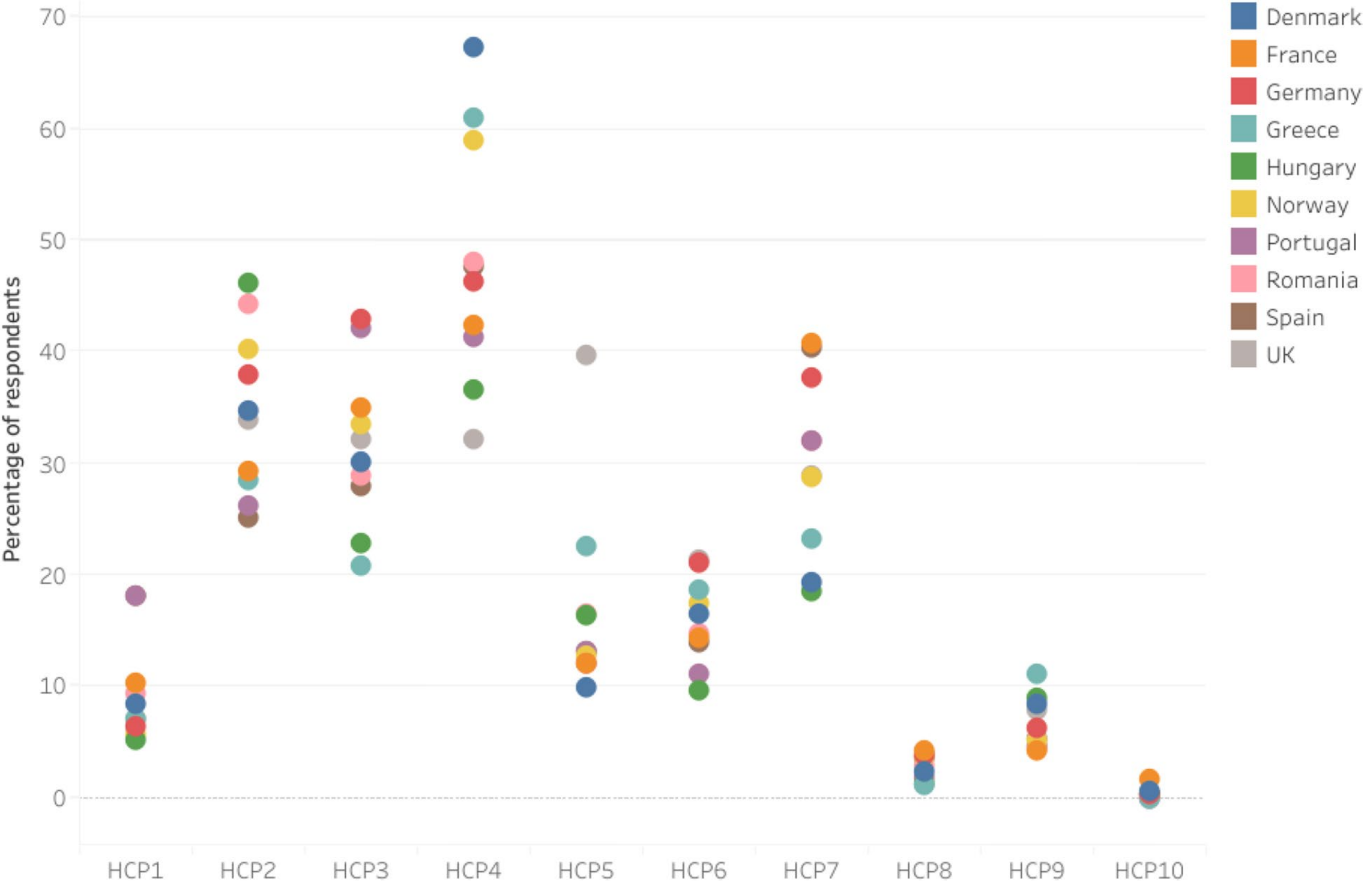
Self-reported hand cleaning methods after touching raw chicken per country based on the SafeConsume survey; only Yes answers are displayed; HCP1 = Wash hands with cold water; HCP2: Wash hands with warm water; HCP3: Wash hands under running water; HCP4: Wash hands with regular soap (bar or liquid); HCP5: Wash hands with antibacterial soap; HCP6: Make sure I wash my hands for at least 20 s; HCP7: Dry hands using a paper towel/cloth/kitchen roll; HCP8: Let my hands dry in the air; HCP9: Disinfect my hands with a hand disinfectant (both alcohol-containing hand sanitizers and sanitizers without alcohol); HCP10: I do not wash my hands; Overlapping bullets indicate a close association regarding respondents’ hand cleaning practices among countries

Figure 6 shows the self-reported hand cleaning methods after touching raw chicken per country based on the SafeConsume survey.

After touching raw chicken, 67.3% of the Danish respondents (549/816) reported washing their hands with regular soap, while this procedure was reported by only 32.1% of the British respondents (294/916). However, the highest frequency of respondents that reported to wash hands for at least 20 s was for the British respondents (21.4%; 196/916), while the lowest percentage was reported for the Hungarian respondents (9.6%; 88/921). The order of the highest percentages regarding proper hand cleaning methods after touching raw chicken based on country reporting was: Denmark, Norway, France, Greece, Hungary, Romania, Spain, UK, Germany, and Portugal. Significant associations were found between countries and proper hygiene practices after handling raw chicken, with the exception of France, Hungary, Spain, and the UK, indicating that French, Hungarian, Spanish, and British respondents are less likely to engage in hand hygiene after handling raw chicken than respondents from the other countries (*p* > 0.05; Table S2).

**Fig. 7:**
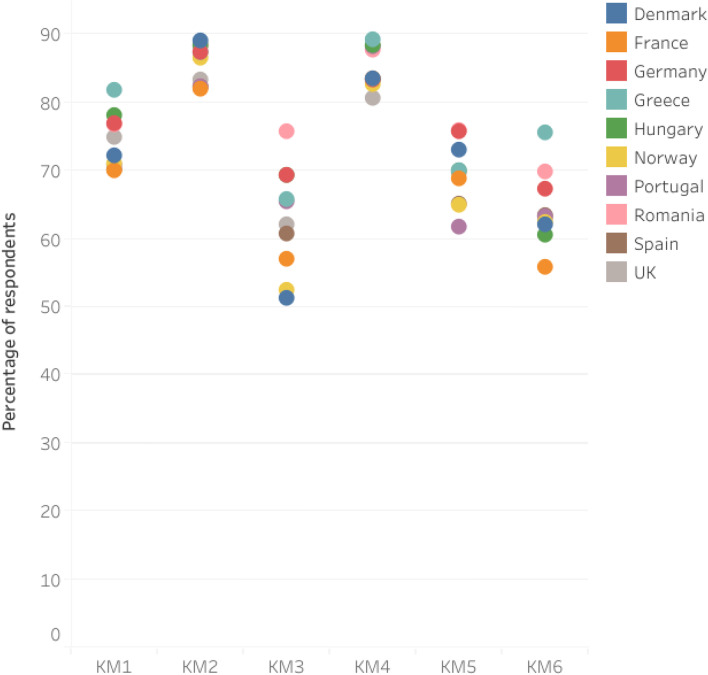
Self-reported hand cleaning methods per country after touching a high-risk item based on the SafeConsume survey; only Yes answers are displayed; KM1 = After touching raw meat or eggs; KM2: After going to the toilet; KM3 = After touching or feeding animals; KM4 = After touching something dirty (physical contact with a soiled/contaminated product/surface); KM5 = After mopping up spillages from poultry or eggs; KM6 = After touching a dirty cloth or sponge; Overlapping bullets indicate a close association regarding respodents’ hand cleaning practices among countries

Figure 7 displays the self-reported hand cleaning methods per country after touching a high-risk item based on SafeConsume survey.

The key moment when most of the respondents declared to wash hands was after going to the toilet (82.2%; 630/767 of the Portuguese respondents to 89.1%; 920/1033 of the Danish respondents). Lower percentages reporting hand washing were observed after touching or feeding animals (from 51.3%; 530/1033 of the Danish respondents to 69.4%; 701/1011 of the Hungarian respondents) and after touching a dirty cloth/sponge (from 55.8%; 560/1005 of the French respondents to 75.6%; 665/880 of the Greek respondents). The order in which the countries were ranked based on the reporting of all key moments when respondents wash their hands was the following: Greece, Romania, Hungary, Germany, Hungary, Denmark, Norway, Portugal, UK, Spain, and France. Significant associations were found for all the countries from this study and key moments when hand washing should occur (*p* < 0.05; Table S3).

**Fig. 8:**
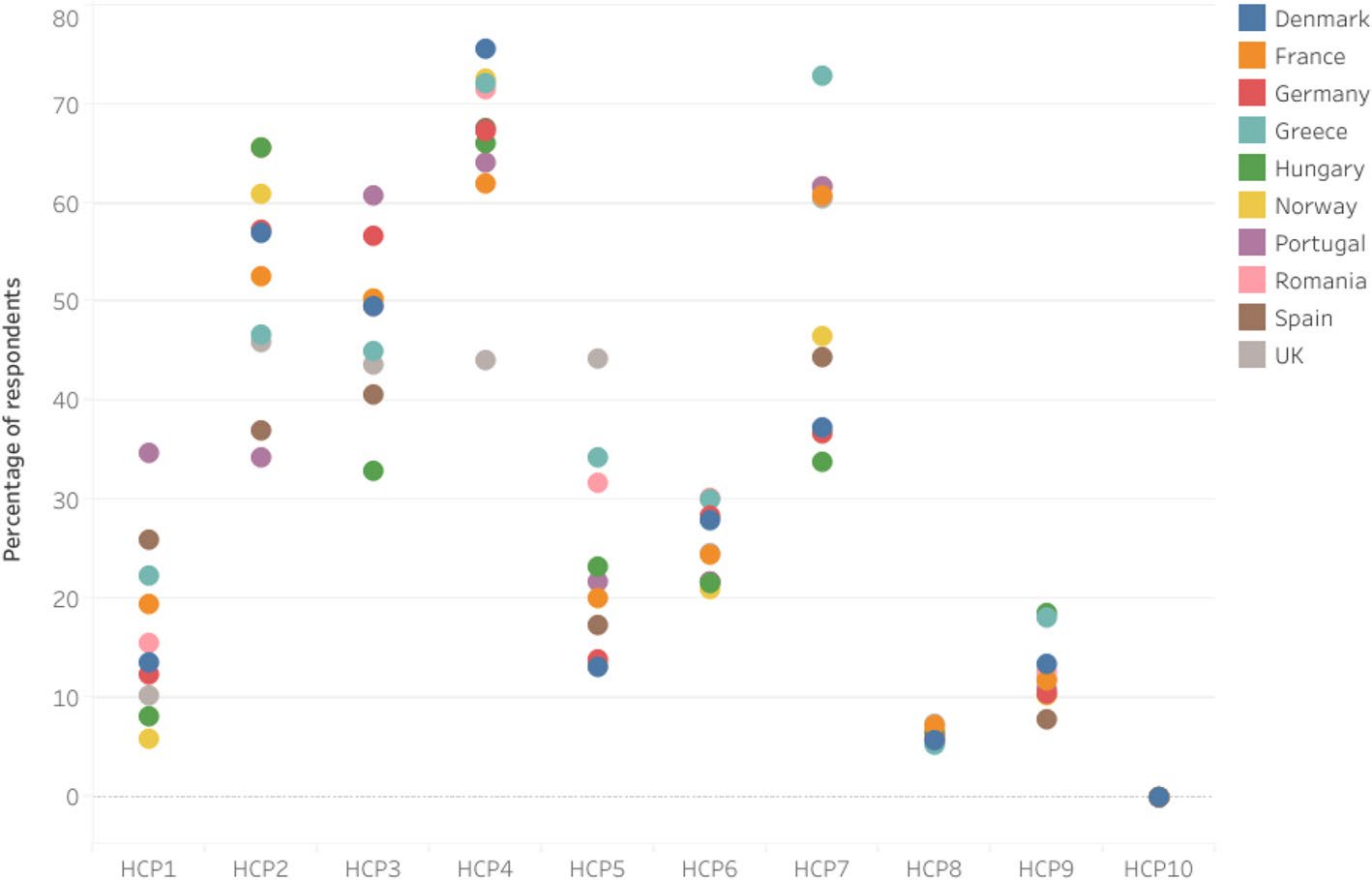
Self-reported general hand cleaning methods per country based on the SafeConsume survey; only Yes answers are displayed; HCP1 = Wash hands with cold water; HCP2: Wash hands with warm water; HCP3: Wash hands under running water; HCP4: Wash hands with regular soap (bar or liquid); HCP5: Wash hands with antibacterial soap; HCP6: Make sure I wash my hands for at least 20 s; HCP7: Dry hands using a paper towel/cloth/kitchen roll; HCP8: Let my hands dry in the air; HCP9: Disinfect my hands with a hand disinfectant (both alcohol-containing hand sanitizers and sanitizers without alcohol); HCP10: I do not wash my hands; Overlapping bullets indicate a close association regarding respondents’ hand cleaning practices among countries

Figure 8 shows the self-reported general hand cleaning methods per country based on SafeConsume survey.

When asked about general hand hygiene practices, 75.5% (780/1033) of Danish respondents reported to wash hands with regular soap, while only 44.1% (476/1080) of the British respondents reported to apply this procedure. Regarding the use of antibacterial soap, the highest percentage was reported by the British respondents (44.2%; 477/1080), Greek respondents (34%; 302/880), and Romanian respondents (31.7%; 312/985). In relation to the duration of hand washing, the highest reported frequencies were from the Romanian respondents (30.3%; 298/985) and Greek respondents (30%; 264/880). We found significant associations between the ten countries and general hand hygiene practices (*p* < 0.05; Table S4) with the exception of the UK (*p* > 0.05), indicating that the British respondents are less likely to engage in safe practices than the respondents from the other nine countries.

The order in which the countries were ranked based on the reported proper hand hygiene practices was the following: Denmark, Norway, Greece, Romania, Spain, Germany, Hungary, Portugal, France, and UK.

Taking into consideration the fact that Denmark had the highest percentages for three out of four questions analysed (“*Likelihood of cleaning hands immediately after touching raw chicken”; “Proper hand cleaning methods after touching raw chicken”*, and “*Proper hand cleaning methods”*) we used it as a reference point when we modelled these questions. Greece had the highest percentages regarding “*Washing hands after touching a high-risk item”* so it was used as a reference point for this question. The goodness of fit tests for the regression models are presented in Table S5. Table 1 shows the regression analysis of respondents’ self-reported hand hygiene practices in relation with their country of origin.

**Table 1:** Regression analysis of respondents’ self-reported hand hygiene practices in relation with their country of origin

Variable	*Likelihood of cleaning hands immediately after touching raw chicken? (N* = *7866)*			
Model 1				
** Country**	**β**	**SE**	**OR (95% CI)**	*p*
France	-1.15	0.99	0.32 (0.26; 0.39)	0.00
Germany	-0.89	0.1	0.41 (0.33; 0.5)	0.00
Greece	-0.29	0.09	0.75 (0.62; 0.91)	0.00
Hungary	-1.01	0.09	0.36 (0.3; 0.44)	0.00
Norway	-0.61	0.09	0.54 (0.45; 0.66)	0.00
Portugal	-0.8	0.1	0.45 (0.37; 0.55)	0.00
Romania	-0.92	0.09	0.4 (0.33; 0.48)	0.00
Spain	-1.03	0.09	0.36 (0.29; 0.43)	0.00
UK	-0.42	0.09	0.65 (0.54; 0.79)	0.00
**Denmark**	0^a^		1	
***Proper hand cleaning methods after touching raw chicken (N*** ** = ** ***7866)***				
** Model 2**				
** Country**	**β**	**SE**	**OR (95% CI)**	*p*
France	-1.02	0.1	0.36 (0.29; 0.44)	0.00
Germany	-0.87	0.1	0.42 (0.34; 0.52)	0.00
Greece	-0.27	0.07	0.76 (0.62; 0.93)	0.00
Hungary	-1.3	0.1	0.28 (0.23; 0.34)	0.00
Norway	-0.36	0.09	0.69 (0.57; 0.85)	0.00
Portugal	-1.08	0.1	0.34 (0.27; 0.42)	0.00
Romania	-0.8	0.1	0.45 (0.37; 0.55)	0.00
Spain	-0.82	0.1	0.44 (0.36; 0.54)	0.00
UK	-1.47	0.1	0.23 (0.19; 0.28)	0.00
**Denmark**	0^a^		1	
***Washing hands after touching a high-risk item (N*** ** = ** ***9966)***				
** Model 3**				
** Country**	**β**	**SE**	**OR (95% CI)**	***p***
Denmark	-0.28	0.04	0.75 (0.6; 0.94)	0.01
France	-0.66	0.1	0.52 (0.41; 0.64)	0.00
Germany	-0.3	0.09	0.73 (0.59; 0.92)	0.00
Hungary	-0.23	0.03	0.79 (0.63; 0.99)	0.03
Norway	-0.610	0.1	0.54 (0.43; 0.67)	0.00
Portugal	-0.61	0.1	0.54 (0.43; 0.67)	0.00
Romania	-0.31	0.09	0.73 (0.58; 0.92)	0.00
Spain	-0.64	0.1	0.52 (0.42; 0.65)	0.00
UK	-0.41	0.09	0.66 (0.53; 0.82)	0.00
** Greece**	0^a^		1	
***Proper hand cleaning methods (N*** ** = ** ***9966)***				
** Model 4**				
** Country**	**β**	**SE**	**OR (95% CI)**	***p***
France	-0.64	0.09	0.53 (0.43; 0.64)	0.00
Germany	-0.4	0.09	0.66 (0.55; 0.8)	0.00
Greece	-0.17	0.07	0.83 (0.68; 1.02)	0.08
Hungary	-0.47	0.09	0.63 (0.52; 0.76)	0.00
Norway	-0.15	0.9	0.85 (0.7; 1.04)	0.11
Portugal	-0.55	0.1	0.58 (0.47; 0.7)	0.00
Romania	-0.21	0.09	0.8 (0.66; 0.98)	0.03
Spain	-0.4	0.1	0.67 (0.55; 0.81)	0.00
UK	-1.36	0.09	0.25 (0.21; 0.3)	0.00
**Denmark**	0^a^		1	

*β* = Regression coefficient, *SE* = Standard Error, *OR (95% CI)* = Odds ratio (95% confidence interval), ^a^reference point, *N* = Number of valid answers, green = “safer than others”; red = fewer safe practices

Regarding the likeliness of washing hands after manipulating chicken with Denmark as the reference country (Table 1; Model 1), respondents from the other nine countries were less likely to wash hands after touching raw chicken than Danish respondents (as indicated by the negative β coefficients and odds ratios < 1; *p* < 0.05; Table 1). Following Denmark, the order of the countries based on the modelling effects was the following: Greece, UK, Norway, Portugal, Germany, Romania, Hungary, Spain, and France (Table 1).

For the self-reported hand cleaning methods after touching chicken with Denmark as the reference point (Table 1; Model 2), all of the other countries were negatively correlated with proper hand hygiene, indicating that the respondents from those countries are less likely to apply adequate hand hygiene than Danish respondents (*p* < 0.05; Table 1). The rank for the correlation between countries and hand washing after handling raw chicken was: Greece, Norway, Romania, Spain, Germany, France, Portugal, Hungary, and the UK.

Modelling hand washing after touching a high-risk item (Table 1; Model 3) showed that respondents from nine countries were less inclined to wash hands at key moments than Greek respondents (*p* < 0.05; Table 1). Following Greece, the order of the countries based on the modelling effects was the following: Hungary, Denmark, Germany, Romania, UK, Norway, Portugal, Spain, and France.

Related to general hand cleaning methods with Denmark as a reference country (Table 1; Model 4), seven of the other countries were negatively correlated with general hand hygiene practices, indicating that the respondents from those countries were less likely to wash hands properly than Danish respondents (*p* < 0.05; Table 1). Two countries, Greece and Norway, were also negatively correlated with general hand hygiene practices, however the effect was not significant (*p* > 0.05; Table 1).

## Discussions

In this study we obtained comparable results to what Italian consumers declare about their engagement in hand washing during cooking (64.5%; 402/624) [16] but different from Brazilian consumers where almost all are self-reporting hand washing during cooking (90.7%; 1103/1217) [29]. However, self-reported practices do not always reflect observed practices. In the study of Mazengia et al., [19] all participants self-reported to wash hands during raw chicken preparation, while the observational study revealed that only 12% washed hands after handling raw poultry. Comparable results were found in other European observational studies where 8/14 British consumers, 3/15 French consumers, 2/12 Portuguese consumers, and 0/15 Romanian consumers washed their hands after touching raw chicken [14].

Similarly, to our results, both European and American consumers exhibit low compliance rates when it comes to washing their hands for 20 s [11, 30], indicating that the recommended duration of hand washing is widely disregarded.

In this study we showed that age, the education level, and inhabitancy are significant predictors of engagement in hand hygiene practices. Age was previously correlated with food safety practices, as indicated by Anderson et al., [31] where consumers > 60 years old are more likely to engage in food safety practices than those < 60 years old. Female consumers were up to two times more likely to report proper hand hygiene practices during raw chicken handling and at key moments (*p* < 0.05; OR = 2.18) [27].

Our findings are also in line with that of Parra et al., [32] who reported that the level of education is correlated with food safety awareness. Previous research showed that Polish consumers that live in cities have a higher level of food hygiene knowledge than those from towns [17].

Although in our study pregnant women were less likely to engage in hand hygiene practices at key moments than non-pregnant women, observational studies revealed that pregnant women are more hygiene cautious due to their care for the safety of their child [14]. Contrary to our results, pregnant women from Slovenia are more likely to wash their hands than non-pregnant women if their hands are dirty [33]. Other studies indicated that pregnant women know how to clean their hands but do not wash them for the recommended duration [15].

Another concern that arrives from this study is that families with elderly members (> > 65 years old) were less likely to apply hand hygiene practices at key moments than families without elderly members. This is of high concern as hands are on the surfaces significantly contaminated in the kitchen [34]. Comparable findings indicate that older adults (> 60 years old) do not engage in hygiene practices [18] and do not wash hands with water and soap during raw chicken handling [34]. These results are further validated by previous research where families with elderly members (> 60 years old) were positively correlated with a higher occurrence of foodborne illnesses [35].

Overall, the regression models indicated that families with vulnerable members are less likely to engage in hand hygiene practices than those without vulnerable members. This is worrisome since vulnerable consumers are more susceptible to foodborne illness and have increased hospitalisation and death rates [36].

Taking into consideration the likelihood of washing hands after touching raw chicken and the percentages scores for proper hand cleaning methods and key moments for hand washing (Figs. 6, 7 and 8) coupled with the regression analysis (Table 1) the rank of the countries regarding proper hand hygiene practices was the following: Denmark, Greece, Norway, Romania, Hungary, Germany, UK, Portugal, France, and Spain.

Monitoring hand washing practices can be obtained directly, e.g., by observation or video recording, or indirectly, e.g., by measuring soap consume or, as in the present study, self-reported surveys. All these different approaches have strengths and weaknesses that are important to be taken into consideration when choosing methodology and when interpreting data. Observational studies are in general more time and financially consuming compared with collecting survey data [37]. The observational studies’ main deficiency is that the observation may influence the practices themselves, e.g., during observation, the hand hygiene rates of consumers increase due to the Hawthorne effect (increased bias) that takes places when consumers are aware of an observer [14]. However, biases may also occur for self-reporting data.

Although observation represents a direct measurement of hand hygiene practices, most studies regarding hand hygiene are self-reported. Overall, self-reported compliance to hand hygiene practices varies but is higher compared to direct observational studies [38].

It is crucial to consider the variance in hygiene information and education across the ten countries included in the study. Differences in cultural practices, socioeconomic factors, and access to education could contribute to divergent behaviours.

Additionally, it is important to explore other factors that might influence these behaviours. For instance, social norms, personal beliefs, and previous experiences with foodborne illnesses could all play a role in shaping individuals' hand hygiene practices.

### Study limitations

The main limitation of this study is that, even though self-reporting is the most efficient way to collect data on a large number of respondents, bias can occur, especially when consumers want to report socially accepted answers (i.e., they are inclined to report a better version of their actual behaviour). To be able to account for washing hands for approximately 20 s it is recommended that consumers sing twice the Happy Birthday song as the average duration of the song is 10 s. Consumers’ practices are not necessarily related to knowledge (some consumers may know what to do but they are honest and admit that they do not do it proper in practice) and they fail to translate their knowledge into practices because of psychological factors, mainly consisting of optimistic bias and habits [39]. Consumers are not aware of their improper food handling practices that could lead to foodborne disease (optimistic bias) and are not able to break out of their risky behaviour [40]. Over-reporting of hand washing is reported to be due to social desirability, recall of information, and dissonance processes [38]. We also did not take into account the combined effect of consuming risky foods such as raw-egg based foods with hand hygiene which can further increase the risk of foodborne disease [41].

## Conclusions

Although washing hands is a skill learnt in early childhood, people practice it in different ways and often skip or shorten the WHO recommended procedure. Even when dealing with risky food like raw chicken, young families with children and/or pregnant women tend to neglect following the complete procedure. Similarly, elderly individuals often overlook the crucial moments for handwashing.

It is worrying that about half of the respondents seem to have insufficient hand washing routines to protect themselves and their family members from foodborne infection.

Significant differences were found between respondents from 10 European countries regarding hand hygiene practices and the overall rank of the countries from the highest to the lowest level of self-reported safe practices was the following: Denmark, Greece, Norway, Romania, Hungary, Germany, UK, Portugal, France, and Spain.

Information and education should point both at the key moments as suggested by the Royal Society for Public Health (RSPH) and the International Scientific Forum on Home Hygiene (IFH) [42] and safe practices.

Further research could delve into identifying the specific barriers and motivations influencing hand hygiene practices among high-risk groups, such as elderly individuals and families with young children. By understanding these factors, interventions and educational campaigns can be designed to effectively promote better hand hygiene practices and mitigate the risks of foodborne illnesses in these vulnerable populations.

Public health burden generated by improper hand washing may be significantly reduced if education is targeted on consumers’ behaviour and practices and specific national behaviours are considered.

## Supplementary Information

**Additional file 1: Table S1. **Kruskal-Wallis test comparing the probability of washing hands after touching raw chicken among 10 European countries.

**Additional file 2: Table S2. **Chi-squared test showing the association between 10 European countries and self-reported proper hand hygiene practicesafter handling raw chicken.

**Additional file 3: Table S3. **Chi-squared test showing the association between countries and self-reported hand washing after touching a high-riskitem.

**Additional file 4: TableS4. **Chi-squaredtest showing the association between countries and general self-reported handcleaning methods.

**Additional file 5: Table S5.** Goodness of fit tests for the regression analysis of consumers’ self-reported hand hygiene practices in relation with their country of origin.

## Data Availability

All of the data used are presented in the manuscript.
