# Supplementary material for: Hand hygiene practices during meal preparation—a ranking among ten European countries
Source: BMC Public Health. 2023 Jul 10;23:1315. doi: 10.1186/s12889-023-16222-5 (PMC10332090; doi:10.1186/s12889-023-16222-5)
Supplement: Supplementary file 1 — Additional file 1: Table S1. Kruskal-Wallis test comparing the probability of washing hands after touching raw chicken among 10 European countries. [file 12889_2023_16222_MOESM1_ESM.docx]

**Table S1.** Kruskal-Wallis test comparing the probability of washing hands after touching raw chicken among 10 European countries

| **How likely is it that you would clean your hands immediately after touching the chicken?** | | | |
| --- | --- | --- | --- |
| **Sample 1-Sample 2** | **Test Statistic** | **SE** | **p** |
| France-Spain | -109.558 | 113.360 | 0.334 |
| France-Hungary | -143.012 | 106.987 | 0.181 |
| France-Romania | -253.381 | 107.686 | 0.019 |
| France-Germany | 302.978 | 116.170 | 0.009 |
| France-Portugal | -406.246 | 118.283 | 0.001 |
| France-Norway | -626.251 | 109.084 | 0.000 |
| France-UK | 809.269 | 107.114 | 0.000 |
| France-Greece | -984.148 | 110.770 | 0.000 |
| France-Denmark | -1220.854 | 109.934 | 0.000 |
| Spain-Hungary | 33.454 | 106.480 | 0.753 |
| Spain-Romania | 143.823 | 107.182 | 0.180 |
| Spain-Germany | 193.421 | 115.703 | 0.095 |
| Spain-Portugal | 296.689 | 117.824 | 0.012 |
| Spain-Norway | 516.693 | 108.587 | 0.000 |
| Spain-UK | 699.712 | 106.607 | 0.000 |
| Spain-Greece | -874.591 | 110.280 | 0.000 |
| Spain-Denmark | -1111.296 | 109.440 | 0.000 |
| Hungary-Romania | -110.369 | 100.418 | 0.272 |
| Hungary-Germany | 159.966 | 109.466 | 0.144 |
| Hungary-Portugal | 263.234 | 111.706 | 0.018 |
| Hungary-Norway | 483.239 | 101.916 | 0.000 |
| Hungary-UK | 666.258 | 99.804 | 0.000 |
| Hungary-Greece | -841.137 | 103.717 | 0.000 |
| Hungary-Denmark | -1077.842 | 102.824 | 0.000 |
| Romania-Germany | 49.597 | 110.149 | 0.653 |
| Romania-Portugal | 152.865 | 112.376 | 0.174 |
| Romania-Norway | 372.870 | 102.649 | 0.000 |
| Romania-UK | 555.888 | 100.553 | 0.000 |
| Romania-Greece | -730.767 | 104.438 | 0.000 |
| Romania-Denmark | -967.473 | 103.551 | 0.000 |
| Germany-Portugal | -103.268 | 120.530 | 0.392 |
| Germany-Norway | -323.273 | 111.517 | 0.004 |
| Germany-UK | 506.291 | 109.590 | 0.000 |
| Germany-Greece | -681.170 | 113.166 | 0.000 |
| Germany-Denmark | -917.875 | 112.348 | 0.000 |
| Portugal -Norway | 220.005 | 113.716 | 0.053 |
| Portugal -UK | 403.023 | 111.828 | 0.000 |
| Portugal -Greece | -577.902 | 115.334 | 0.000 |
| Portugal -Denmark | -814.607 | 114.531 | 0.000 |
| Norway-UK | 183.018 | 102.049 | 0.073 |
| Norway-Greece | -357.897 | 105.879 | 0.001 |
| Norway-Denmark | -594.602 | 105.005 | 0.000 |
| UK-Greece | -174.879 | 103.848 | 0.092 |
| UK-Denmark | -411.584 | 102.956 | 0.000 |
| Greece-Denmark | -236.705 | 106.754 | 0.027 |

SE = standard error; *p* significant at < 0.05
