# Supplementary material for: Hand hygiene practices during meal preparation—a ranking among ten European countries
Source: BMC Public Health. 2023 Jul 10;23:1315. doi: 10.1186/s12889-023-16222-5 (PMC10332090; doi:10.1186/s12889-023-16222-5)
Supplement: Supplementary file 2 — Additional file 2: Table S2. Chi-squared test showing the association between 10 European countries and self-reported proper hand hygiene practicesafter handling raw chicken. [file 12889_2023_16222_MOESM2_ESM.docx]

**Table S2.** Chi-squared test showing the association between 10 European countries and self-reported proper hand hygiene practices after handling raw chicken

| **Proper hand hygiene methods after handling raw chicken** | | |
| --- | --- | --- |
| **Country** | | |
| Denmark | Chi-square | 97.456 |
|  | df | 1 |
|  | p | 0.000 |
| France | Chi-square | 15.915 |
|  | df | 1 |
|  | p | 0.061 |
| Germany | Chi-square | 3.534 |
|  | df | 1 |
|  | p | 0.060 |
| Greece | Chi-square | 38.324 |
|  | df | 1 |
|  | p | 0.000 |
| Hungary | Chi-square | 66.242 |
|  | df | 1 |
|  | p | 0.08 |
| Norway | Chi-square | 26.659 |
|  | df | 1 |
|  | p | 0.000 |
| Portugal | Chi-square | 18.800 |
|  | df | 1 |
|  | p | 0.074 |
| Romania | Chi-square | 1.450 |
|  | df | 1 |
|  | p | 0.229 |
| Spain | Chi-square | 1.610 |
|  | df | 1 |
|  | p | 0.12 |
| UK | Chi-square | 117.450 |
|  | df | 1 |
|  | p | 0.204 |

df = degrees of freedom; *p* significant at < 0.05
