# Supplementary material for: Hand hygiene practices during meal preparation—a ranking among ten European countries
Source: BMC Public Health. 2023 Jul 10;23:1315. doi: 10.1186/s12889-023-16222-5 (PMC10332090; doi:10.1186/s12889-023-16222-5)
Supplement: Supplementary file 3 — Additional file 3: Table S3. Chi-squared test showing the association between countries and self-reported hand washing after touching a high-riskitem. [file 12889_2023_16222_MOESM3_ESM.docx]

**Table S3.** Chi-squared test showing the association between countries and self-reported hand washing after touching a high-risk item

| **Hand hygiene practices after touching a high-risk item** | | |
| --- | --- | --- |
| **Country** | | |
| Denmark | Chi-square | 304.667 |
|  | df | 1 |
|  | p | 0.000 |
| France | Chi-square | 160.001 |
|  | df | 1 |
|  | p | 0.000 |
| Germany | Chi-square | 295.410 |
|  | df | 1 |
|  | p | 0.000 |
| Greece | Chi-square | 356.364 |
|  | df | 1 |
|  | p | 0.000 |
| Hungary | Chi-square | 317.991 |
|  | df | 1 |
|  | p | 0.000 |
| Norway | Chi-square | 177.022 |
|  | df | 1 |
|  | p | 0.000 |
| Portugal | Chi-square | 162.188 |
|  | df | 1 |
|  | p | 0.000 |
| Romania | Chi-square | 281.958 |
|  | df | 1 |
|  | p | 0.000 |
| Spain | Chi-square | 165.789 |
|  | df | 1 |
|  | p | 0.000 |
| UK | Chi-square | 268.004 |
|  | df | 1 |
|  | p | 0.000 |

df = degrees of freedom; *p* significant at < 0.05
