# Supplementary material for: Hand hygiene practices during meal preparation—a ranking among ten European countries
Source: BMC Public Health. 2023 Jul 10;23:1315. doi: 10.1186/s12889-023-16222-5 (PMC10332090; doi:10.1186/s12889-023-16222-5)
Supplement: Supplementary file 4 — Additional file 4: TableS4. Chi-squaredtest showing the association between countries and general self-reported handcleaning methods. [file 12889_2023_16222_MOESM4_ESM.docx]

**Table S4.** Chi-squared test showing the association between countries and general self-reported hand cleaning methods

| **Proper hand cleaning methods** | | |
| --- | --- | --- |
| **Country** | | |
| Denmark | Chi-square | 268.857 |
|  | df | 1 |
|  | p | 0.000 |
| France | Chi-square | 56.837 |
|  | df | 1 |
|  | p | 0.000 |
| Germany | Chi-square | 122.379 |
|  | df | 1 |
|  | p | 0.000 |
| Greece | Chi-square | 171.073 |
|  | df | 1 |
|  | p | 0.000 |
| Hungary | Chi-square | 101.920 |
|  | df | 1 |
|  | p | 0.000 |
| Norway | Chi-square | 203.085 |
|  | df | 1 |
|  | p | 0.000 |
| Portugal | Chi-square | 73.013 |
|  | df | 1 |
|  | p | 0.000 |
| Romania | Chi-square | 179.940 |
|  | df | 1 |
|  | p | 0.000 |
| Spain | Chi-square | 122.102 |
|  | df | 1 |
|  | p | 0.000 |
| UK | Chi-square | 15.170 |
|  | df | 1 |
|  | p | 0.19 |

df = degrees of freedom; *p* significant at < 0.05
