# Supplementary material for: Hand hygiene practices during meal preparation—a ranking among ten European countries
Source: BMC Public Health. 2023 Jul 10;23:1315. doi: 10.1186/s12889-023-16222-5 (PMC10332090; doi:10.1186/s12889-023-16222-5)
Supplement: Supplementary file 5 — Additional file 5: Table S5. Goodness of fit tests for the regression analysis of consumers’ self-reported hand hygiene practices in relation with their country of origin. [file 12889_2023_16222_MOESM5_ESM.docx]

**Table S5.** Goodness of fit tests for the regression analysis of consumers’ self-reported hand hygiene practices in relation with their country of origin

|  | Omnibus Test | Pearson Test | Deviance Test | Test of Parallel Lines |
| --- | --- | --- | --- | --- |
| Model 1 | χ²(9) = 273.576,  *p* = 0.000 | χ²(1444) = 1407.63,  *p* = 0.68 | χ²(1444) = 1219.71,  *p* = 0.9 | *p* = 0.34 |
|  | **Omnibus Test** | **Hosmer and Lemeshow Test** | |  |
| Model 2 | χ²(7) = 382.120,  *p* = 0.000 | χ²(8) = 11.851, *p* = 0.57 | |  |
| Model 3 | χ²(7) = 29.632,  *p* = 0.000 | χ²(8) = 75.643, *p* = 0.58 | |  |
| Model 4 | χ²(7) = 318.740,  *p* = 0.000 | χ²(8) = 11.348, *p* = 0.87 | |  |
